# Finger (phalangeal) hair as a donor source for eyebrow restoration

**DOI:** 10.1016/j.jdcr.2026.06.064

**Published:** 2026-07-07

**Authors:** Artur Diaz Carandell, Natacha Alavi, Maxime Nivard, Maximo Mignone, Roy Argueta

**Affiliations:** Hair Transplant Surgery, Clinique Racine Carrée, Paris, France

**Keywords:** body hair transplantation, digital hair, donor site, eyebrow alopecia, eyebrow restoration, eyebrow transplantation, finger hair, follicular unit extraction, FUE, phalangeal hair

## Introduction

Eyebrow alopecia has multiple etiologies, including alopecia areata,^1^ frontal fibrosing alopecia,^2^^,^^3^ inflammatory or traumatic conditions, mechanical causes such as trichotillomania and chronic over-plucking, nutritional deficiencies, autoimmune disease, and chemotherapy.^4^ When pharmacological options are insufficient, surgical hair restoration is well established.^5^ The standard donor area is occipital or periauricular scalp; however, its long anagen phase causes continuous post-transplant growth requiring regular trimming.^6^ Alternative donors proposed to improve morphological compatibility with native eyebrow hair include leg,^7^ fronto-temporal triangle scalp,^8^ and beard hair, although the latter shares the long anagen pattern of scalp hair and additionally requires routine styling. Finger (phalangeal) hair, in contrast, has a short anagen phase and limited natural length, theoretically matching native eyebrow hair dynamics. We present, to our knowledge, the first such case.

## Case report

**Fig 1:**
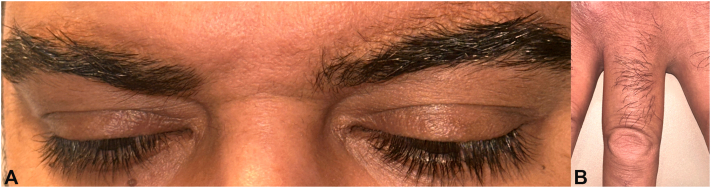
Pre-operative status. **A,** Partial bilateral eyebrow hypotrichosis with hypodensity of the upper third of both eyebrows. **B,** Proximal phalangeal donor hair, showing fine caliber, texture, and limited natural length comparable to native eyebrow hair.

A 35-year-old man presented with partial bilateral eyebrow hypotrichosis after years of cosmetically motivated over-plucking. The behavior was conscious and episodic, did not meet DSM-5 criteria for trichotillomania,^9^ and had ceased prior to surgical planning. Hypodensity was noted in the upper third of both eyebrows (Fig 1, *A*). Scalp- and beard-based donors were declined by the patient because of the trimming and styling burden their long anagen phase entails. Leg hair was clinically assessed as a potential donor but discarded because the patient’s leg hair was sparse and discordant in caliber from native eyebrow hair. The dorsum of the proximal phalanges showed hair matching native eyebrow hair in caliber, texture, and natural length (Fig 1, *B*); FUE harvest of finger hair was therefore proposed. Written informed consent was obtained for the procedure and publication.

**Fig 2:**
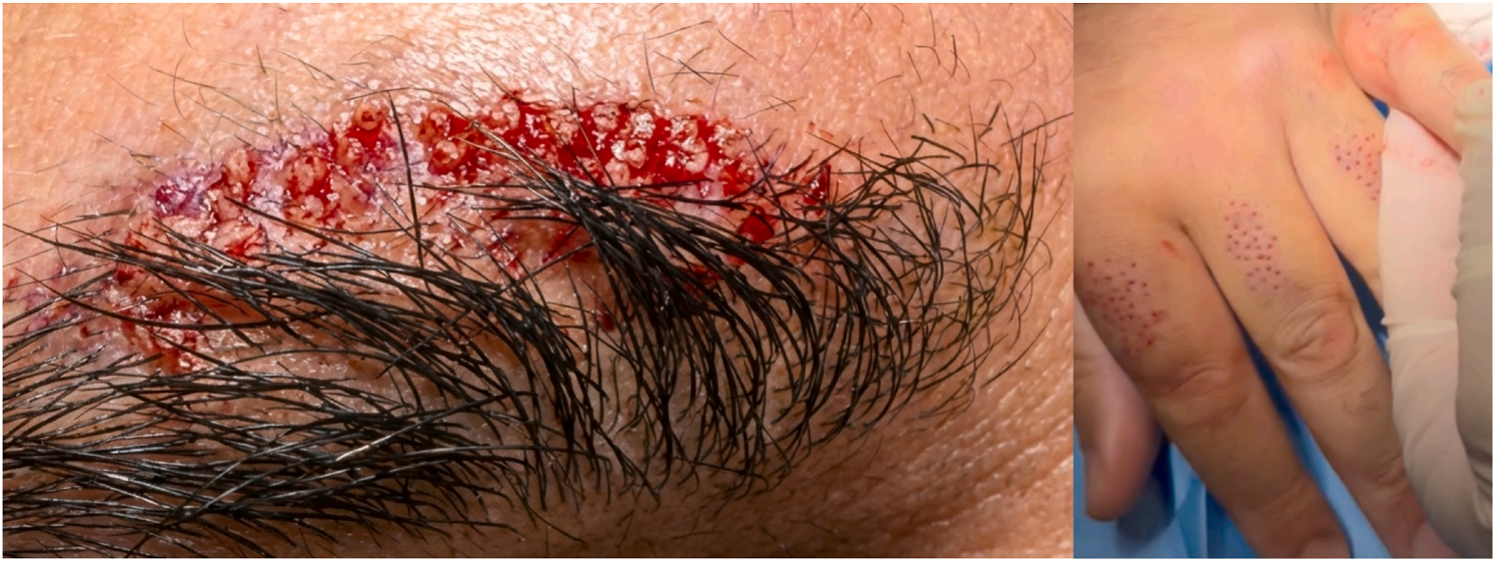
Donor-site harvest. Distribution of follicular unit extraction sites across the dorsum of the proximal phalanges, distributed evenly to minimize localized depletion. Recipient sites filled with grafts.

The procedure was performed under local anesthesia with prophylactic oral amoxicillin 500 mg every 8 hours started the day before surgery. A dorsal digital nerve block was performed in each finger,^10^ using 0.2 mL per finger of a solution containing 100 mL saline, 100 mg lidocaine 1%, and 0.5 mg epinephrine (1:200,000). Tumescent infiltration with the same solution followed at the donor sites. A 0.7-mm serrated motorized FUE punch was used to extract single follicular units from the dorsum of the proximal phalanges of all 10 fingers (Video 1, available on www.jaad.org, Fig 2), distributed evenly to minimize localized depletion. A total of 120 single-hair grafts were harvested and stored in chilled Ringer’s lactate.

Recipient sites were created bilaterally with a 1.3-mm sapphire blade, replicating native exit angles, direction, and density variation. Recipient anesthesia used the same solution by infiltration; only minimal, transient periocular edema was observed and resolved within 24 hours. Grafts were placed by forceps. Operative time was 3 hours, with no intraoperative complications. Donor area postoperative care consisted of topical mupirocin twice daily. Oral amoxicillin for 4 additional days, ibuprofen 600 mg every 8 hours for 2 days, and omeprazole 20 mg daily for 5 days; no occlusive dressings were used.

**Fig 3:**
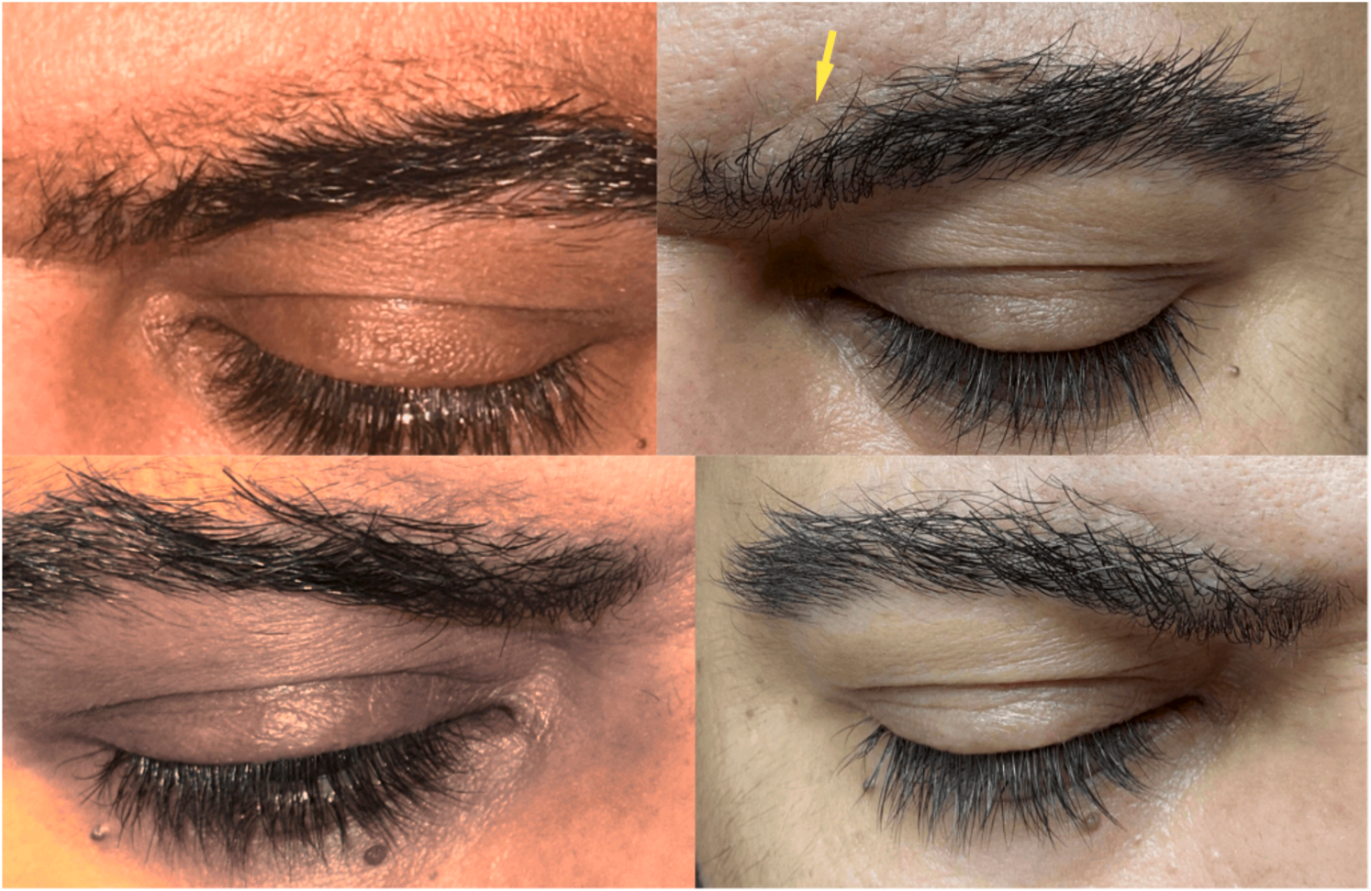
Eight-month postoperative result of the recipient area. Satisfactory partial restoration of bilateral eyebrow density following finger hair FUE, with a small area of incomplete regrowth (*arrow*).

**Fig 4:**
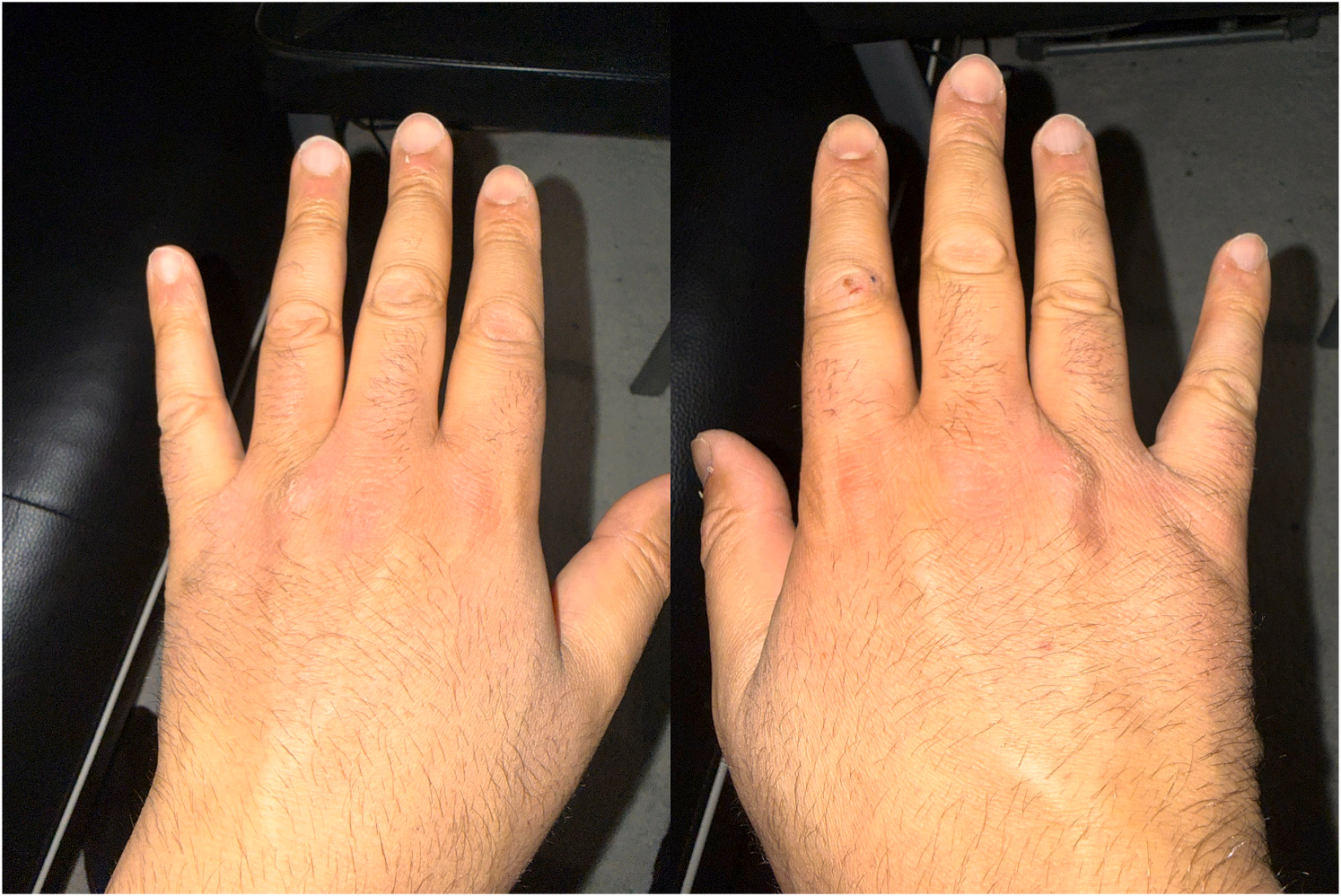
Eight-month postoperative result of the donor area. Dorsum of the proximal phalanges showing absence of visible scarring or cosmetic compromise at the harvest sites.

Final assessment was at 8 months, the longest available follow-up. Bilateral density was partially and satisfactorily restored, with successful growth across most of the treated area (Fig 3). A small region of incomplete regrowth was observed; whether this represents delayed post-transplant telogen effluvium with potential for further recovery, or graft failure, cannot be distinguished at this time point. Transplanted hairs grew with limited length not requiring trimming. No donor- or recipient-site complications occurred (Fig 4). The patient expressed satisfaction and requested a secondary procedure to address the area of incomplete coverage.

## Discussion

To our knowledge, this is the first reported use of finger hair as a donor source for eyebrow transplantation by FUE. The rationale rests on morphological compatibility: finger hair shares the fine caliber and limited growth length of native eyebrow hair, properties that scalp and beard hair do not.^11^ By the principle of donor dominance, transplanted hairs retain donor growth characteristics^12^; finger-derived grafts are therefore expected to remain short. The absence of any required trimming over 8 months supports this expectation, in contrast with the maintenance burden described after scalp-based eyebrow restoration. Table I summarizes the principal donor sources reported for eyebrow restoration; in our case both scalp and beard were declined on maintenance grounds.

**Table I:** Donor sources reported for eyebrow restoration, with comparative growth characteristics

Donor source	Caliber	Anagen/growth	Trimming	Reported use
Occipital/periauricular scalp	Medium–coarse	Long; continuous	Frequent	Standard donor^5^^,^^6^
Beard	Coarse	Long; continuous + styling	Frequent + styling	Reported in men
Frontal-temporal triangle	Fine	Intermediate	Occasional	Asian patients^8^
Leg	Fine	Short; limited length	Minimal	Body hair FUE^7^
Finger (proximal phalanx)	Fine	Short; limited length	None at 8 months	Present case

Technically, the flat dorsal anatomy of the proximal phalanx facilitated motorized punch use, and the 0.7-mm punch produced no visible donor scarring at 8 months. Distributing extractions across all 10 fingers limited depletion at any single site, analogous to body hair FUE strategies.^7^ Topical minoxidil or bimatoprost act on viable follicles and have limited expected effect when follicles have been physically removed by chronic mechanical avulsion; surgical replacement was therefore considered first-line.

The principal limitation is graft yield: finger hair density is lower than scalp or leg, and the maximum harvest obtainable from the 10 proximal phalanges has not been characterized in the literature. In our experience, finger hair is best suited to partial eyebrow alopecia with low follicular demand (in the order of <200 FU), or as a complementary source. The aggregate donor yield remains an open question and a relevant line of future investigation. Hair on the dorsum of the hand and on the dorsum of the proximal phalanges of the toes shares some of the morphological features that motivated finger hair selection (fine caliber, short anagen) and constitutes anatomically plausible alternative donor regions; these sites were not systematically evaluated in the present case and a direct comparative assessment of caliber, density, and yield across phalangeal, dorsal hand, and toe hair represents a further line of investigation. As visible in Fig 4, dorsal hand hair was present in this patient and was not formally compared with phalangeal hair in caliber or density. The proximal phalanx of the finger was preferred in our case on practical grounds: a flat dorsal surface favorable to motorized punch use, accessible patient and surgeon positioning, and 10 anatomically discrete harvest sites allowing distribution of extractions to minimize localized depletion. The report is additionally limited by its single-case design, 8-month follow-up, partial success, and absence of quantitative graft survival metrics. The etiology in this case—prolonged cosmetically motivated over-plucking that does not meet trichotillomania criteria—represents a distinct entity; when the extraction pattern suggests a compulsive component, surgeons should consider psychological screening even if formal DSM-5 thresholds are not reached, given the risk of recurrent self-extraction of transplanted hairs.

## Conclusions

Finger (phalangeal) hair is a previously unreported and anatomically plausible donor source for eyebrow restoration by FUE in selected patients with low follicular demand who decline scalp- or beard-based donors. Further investigation is warranted to characterize maximum donor yield and define optimal patient selection.

## Conflicts of interest

None disclosed.

## Supplementary data

Video E1
